# Remote learning slightly decreased student performance in an introductory undergraduate course on climate change

**DOI:** 10.1038/s43247-022-00506-6

**Published:** 2022-08-06

**Authors:** Sattik Ghosh, Stephanie Pulford, Arnold J. Bloom

**Affiliations:** 1 Center for Educational Effectiveness, University of California, Davis, CA USA; 2 School of Education, University of California, Davis, CA USA; 3 Department of Plant Sciences, University of California, Davis, CA USA; 4 Present Address: Tempo Automation, 2460 Alameda Street, San Francisco, CA 94103 USA

**Keywords:** Science in culture, Climate change

## Abstract

Public understanding about complex issues such as climate change relies heavily on online resources. Yet the role that online instruction should assume in post-secondary science education remains contentious despite its near ubiquity during the COVID-19 pandemic. The objective here was to compare the performance of 1790 undergraduates taking either an online or face-to-face version of an introductory course on climate change. Both versions were taught by a single instructor, thus, minimizing instructor bias. Women, seniors, English language learners, and humanities majors disproportionately chose to enroll in the online version because of its ease of scheduling and accessibility. After correcting for performance-gaps among different demographic groups, the COVID-19 pandemic had no significant effect on online student performance and students in the online version scored 2% lower (on a scale of 0–100) than those in the face-to-face version, a penalty that may be a reasonable tradeoff for the ease of scheduling and accessibility that these students desire.

## Abstract (web summary)

Students who completed the online version of an introductory course on climate change performed 2% worse than those who completed the in-person version, according to a study of 1790 undergraduate students in California, USA.

## Introduction

Support for policies that address climate change depends on an educated populace and its comprehension of difficult scientific concepts. To forestall action on climate change, the government of the United States in 2017 removed hundreds of webpages about climate change from the websites of federal agencies and departments and scrubbed the term “climate change” from thousands of others. Only four years later after a new administration took office was this censoring reversed. Also troubling is that during this period some reliable sources of information became less suitable for educational purposes; for example, the Assessment Reports of the United Nations Intergovernmental Panel on Climate Change (IPCC) grew exponentially: the reports for Working Group I about the Physical Science expanded from 414 pages in 1990 to 3949 pages in 2021, for Working Group II about Impact and Adaptation from 296 pages in 1990 to 3675 pages in 2021, and for Working Group III about Mitigation from 438 pages in 1995 to 2913 pages in 2022 (Fig. S1). To address these issues, the National Science Foundation of the United States, as part of DUE 09-50396 “Creating a Learning Community for Solutions to Climate Change”, funded establishment of a nationwide cyber-enabled learning community to develop web-based curricular resources for teaching undergraduates about climate changes. One product of this project was a multi-disciplinary, introductory online course that is freely available to the public^1^.

This course was pressed into broader service as schools struggled to provide online materials at the onset of the COVID-19 pandemic. Institutions of higher education received criticism for adopting such courses, largely based on the assumption that online instruction is inherently inferior to that delivered face-to-face. The issue has become whether the convenience and safety of online instruction outweighs the possibility of inferior learning outcomes for today’s undergraduates.

Although the pandemic infused topical urgency into this issue, it is hardly new. The efficacy of distance learning has been debated since the External Programme of the University of London first offered a correspondence course in 1858. Correspondence degrees have historically been driven by equity concerns for working people and women who could not access colleges^2^, yet they have historically been perceived as inferior to on-campus education^3,4^.

Online learning opportunities experienced explosive growth with the advent of widespread internet access and expanded credentialed university programs. In the United States alone, enrollments in online college courses rose from 1.6 million students in 2002 to 6.9 million students in 2018^5,6^. During 2018, 35.3% of undergraduates in the United States took at least one course online, and half of these students took online courses exclusively^6^. This boom in online offerings coevolved with active learning and EdTech, and today’s online courses tend to be highly interactive, even when asynchronous or self-paced. Indeed, instructional design proponents often position today’s online courses on a spectrum with hybrid learning and flipped classrooms, rather than emphasize their ascent from didactic-style correspondence courses. Advocates for interactive online learning claim that a well-designed online course can be as effective as a face-to-face course, and perhaps even more effective than a traditional course based on passive lecture presentations^7–10^.

Despite the new pedagogic paradigm for today’s online courses, familiar critiques of online learning persist^4^. Detractors cite high attrition rates as evidence that online courses leave students vulnerable to distraction and claim that the quality of educational experience and achievement in an online course cannot match that of a similar face-to-face class. Compounding these critiques, a number of studies in higher education have suggested that online courses, like their historic distance-education counterparts, tend to disproportionately enroll underserved students: if these already-vulnerable students are being attracted to a lower-quality educational experience online, then the proliferation of these courses might constitute an educational trap, exacerbating achievement gaps and providing barriers to persistence and success^11,12^.

The efficacy of online versus face-to-face courses seems ripe for an evidence-based study, yet high quality pseudo-experiments that compare the efficacies remain elusive. For example, The U.S. Department of Education in 2010 conducted a meta-analysis of 28 studies comparing online versus face-to-face learning in post-secondary education settings and concluded, “When used by itself, online learning appears to be as effective as conventional classroom instruction, but not more so”^13^. A re-evaluation of this meta-analysis, however, found only four of these studies used an appropriate experimental design and examined semester-length college courses: in three of the studies, the students in the online versions of a course had poorer outcomes than those in the face-to-face versions, whereas in the fourth study, the students in the two versions had roughly similar outcomes^14^.

More recently, several large-scale studies of college students in the United States determined that student outcomes—both persistence through the end of the course and final grades—were substantially poorer for online courses than for face-to-face courses^15–24^. These studies, however, were based on comparisons of courses with either different subject matter, those taught by different instructors, or those having relatively small numbers of students. Because many of these studies are based on dissimilar courses, they have had no opportunity to isolate students’ enrollment decisions to a simple choice between an online and a face-to-face version, nor provide appropriate analysis to account for the potential effects of underserved groups’ preference for one format over the other.

It follows that prior pseudo-experimental studies have also been unable to examine the critical concern that underlies all comparisons of online and face-to-face courses: if a tradeoff does exist between a face-to-face course’s baseline educational outcomes and an online course’s extended accessibility, is the decrease in learning outcomes worth the attendant increase in accessibility? These tradeoffs have been imbued with new urgency because of the COVID-19 pandemic during which universities and students seek to make difficult decisions about how to ensure safe course access while optimizing learning outcomes during the disruption of unfettered public life.

**Fig. 1:**
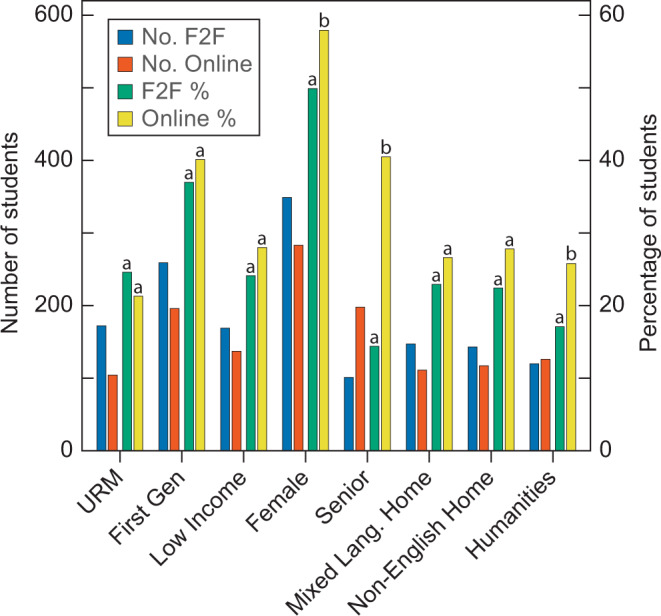
Number (No.) or percentage (%) of students with a particular self-identified trait enrolled in Face-to-Face (F2F) and Online versions during Winter quarters before the pandemic for an introductory, undergraduate course on climate change. “No. F2F” and “No. Online” are the numbers of students who were underrepresented minorities (URM) (African Americans, American Indian/Alaska Native, Chicanx/Latinx including Puerto Rican, and Pacific Islander including Native Hawaiian), first generation college student (First Gen), student with an annual family income of less than $80,000 (Low Income); student in their last year of college (Senior); student majoring in a humanities discipline (Humanities); “F2F %” and “Online %” are the percentages of students with a trait. Different letters above the bars indicate that % of students with a trait differed significantly (*P* < 0.05) between the F2F and online versions.

In this study, we seek to dissect student choice, student outcomes, and the tradeoffs between online and face-to-face courses at a large research university, through a post-hoc pseudo-experiment. We analyzed student performance versus their attributes for 1790 undergraduates of the University of California at Davis (a public research university) who enrolled in either an online or face-to-face version of the introductory course about climate change (for a syllabus of the course see Table 1). Each demographic group had more than 100 students enrolled in the online and face-to-face versions (Fig. 1). Each year, both versions of the course were taught by a single instructor, thus, minimizing major confounding variables such as instructor bias, course design, content differences, and other aspects that might influence student choices and outcomes. Before the COVID-19 pandemic, we offered both versions of the course during eight Winter quarters and offered only the online version during six Spring quarters. In Winter and Spring quarters 2021, during the pandemic, we offered the course only online. For two concurrent course offerings in Winter 2019—one face-to-face and one online—and for COVID-19 pandemic-induced online course offerings in Winter 2021 and Spring 2021, we surveyed the students about their past experiences with online learning and how these experiences influenced their choice between the online and face-to-face versions of the course.

**Table 1:** Syllabus: global climate change SAS 25 (face-to-face) and 25v (online).

4 Credit Units, No Prerequisites; General Education Credits for Science & Engineering, Social Sciences, Domestic Diversity, Writing, Oral Presentations, Quantitative Skills, Scientific Literacy, Visual Literacy, and World Cultures					
**Activity**		**Hours per week**			
Readings		2.5			
Lectures: Live and Mini		3.0			
Discussion		1.0			
Quiz		1.5			
Exercise or Essay		4.0			
Total		12.0			
Textbook: Climate Change: Causes, Consequences, and Solutions. Free online at https://indd.adobe.com/view/7eafc24d-9151-4493-85d2-cb3f2e5a2a51. Please read the simple directions on navigating through this textbook at *How to Dance*.					
SAS 25v also requires: A headset (any combination of headphones and microphone), a webcam, high speed, reliable connection to the internet (DSL, cable, on-campus, etc.)					
**Week**	**Topic**		**Video**	**Reading**	**Assignments**
1	Intro to climate research		Lecture 1–3	Chapt. 1	Exercise 1
2	History of Earth’s climate		Lecture 4–7	Chapt. 2	Essay 1
3	Causes of climate		Lecture 8–11	Chapt. 3	Exercise 2
4	Climate models		Lecture 12–17	Chapt. 4	Essay 2
5	Climate & biosphere		Lecture 18–22	Chapt. 5 & 6	Exercise 3 Midterm
6	Transportation		Lecture 23–27	Chapt. 7	Essay 3
7	Electricity & other sectors		Lecture 28–36	Chapt. 8 & 9	Exercise 4
8	Climate change economics		Lecture 37–42	Chapt. 10	Essay 4
9	Environmental law		Lecture 43–46	Chapt. 11	Exercise 5
10	Culture & climate change		Lecture 47–48	Chapt. 12	Essay 5, Final
Exercise Assignments: These should prepare you for writing the essay assignments. You will have one learning exercise due every other week and we will review your answers during discussion sections. These are typically 2 to 3 pages in length.					
**Week**	**Topic**				
1	Climate Trends: Examine temperature graphs for climate change trends in Davis, CA				
3	How to Read a Scientific Article: Critically read and summarize a scientific article on a GCM				
5	Climate Change and Species: Discover how polar bears are affected by climate change				
7	Carbon Footprint: Calculate your contributions to GHG emissions				
9	Countries and Climate Change: Discover how the culture of the USA influences climate change actions				
Essay Assignments: The essays are typically 2 to 3 pages in length.					
**Week**	**Topic**				
2	Climate Trends in Your Hometown: Create temperature graphs for your hometown using ground station and satellite sources. Discuss temperature trends and running averages				
4	Global Climate Model: Use the library to research a GCM and discuss that GCM including history, how it works, and why it is important				
6	Climate Change and Species: Choose a species and discuss how that species will respond to climate change				
8	Carbon Footprint: Reduce your GHG emissions by 50% for one day and discuss the experience including costs vs. benefits of doing so				
10	Countries and Climate Change: Choose a country and discuss how its culture influences climate change actions				
**Grading:**					
5	Exercises (each 3% of the grade for a total of 15%)				
5	Essays (each 8% of the grade for a total of 40%); deduct 5% of the grade per day for late assignments				
10	Online Quizzes: one per week (each 1% of the grade for a total of 10%)				
1	Midterm: 25 Multiple choice questions and an essay about the greenhouse effect (10%)				
1	Final: 50 Multiple choice questions and an essay about what we should do, if anything, to address climate change (15%)				
10	Discussion section presentations and participation (each 1% of the grade for a total of 10%)				

Causes of global climate change and the biological, geophysical, and social consequences of such change. Methods used by different scientists for predicting future events. Complexity of global affairs. Decision making under uncertainty.

All elements of the course are available for free at https://www.climatechangecourse.org/, including a free multi-media textbook at https://indd.adobe.com/view/7eafc24d-9151-4493-85d2-cb3f2e5a2a51 that is updated regularly. During the period from 2017 to 2021, the online textbook had 5000 new users per year, who each averaged at least 3 views and 10 min per view. Before 2017, a printed version of the textbook was available for purchase^25^.

## Results

Before the COVID-19 pandemic (2013 through 2020), we taught both the online and face-to-face versions of the course concurrently during Winter quarters and only the online version during most Spring quarters. During the pandemic in Winter and Spring quarters 2021, we taught only the online version of the course. We found no significant difference in the grades for students enrolled in the online version before and during the pandemic (Table S1); therefore, in a subsequent analysis that compared the grades between the online and face-to-face versions, we merged the data for Winter and Spring 2021 with earlier data from the online version from 2013 to 2020 (Table 2).

**Table 2:** Regressions of course grade in an introductory, undergraduate course on climate change for all quarters.

Variable	Model 1	Model 2	Model 3
Intercept	86.48***	87.83***	52.89***
Online	–2.07***	–1.99***	–2.00***
Mixed Lang. Home		–0.12	1.98**
Non-English Home		–0.96	–0.02
Male		–0.75	–0.12
Senior		–1.11	–1.90**
Humanities		–4.91***	–5.80***
GPA			11.54***
URM			–4.09***
Low Income			–0.98
First Gen			1.34*

“Intercept” predicts the average grade of students in the face-to-face version on a 0–100 scale. The three models include Model 1 where “Online” (0 or 1) is the influence of the online version on a student’s grade. Model 2 adds the influence of other Languages being spoken at home, self-identifying as Male, being a Senior, and majoring in Humanities (all 0 or 1). Model 3 adds the influence of the student’s GPA (grade point average between 0 and 4), being an Underrepresented Minority (0 or 1), being from a Low-Income family (0 or 1) (annual family income of less than $80,000), and being the First Generation to attend college (0 or 1). Asterisks following a number indicates *P* values associated with *t* values for the Wald test of the hypothesis H_0_:*β*_i_ = 0: “*” indicates *P* < 0.05, “**” *P* < 0.01, and “***” *P* < 0.001.

Overall, students performed poorer in the online version. Humanities students, Underrepresented Minorities (i.e., African Americans, American Indian/Alaska Native, Chicanx/Latinx including Puerto Rican, and Pacific Islander including Native Hawaiian), and Seniors (i.e., students in their last year) received significantly lower grades than other students enrolled in either the online or face-to-face formats (Table 2). The factor that consistently had the largest influence on a student’s grade in this course was the student’s overall Grade Point Average (GPA) (Table 2, S1, S2, S3 and S5), demonstrating that students, who on average performed well in all their courses, performed well in this course. Students who spoke Mixed Languages at Home and those who were the First Generation to attend college received slightly higher grades than other students. Students from Low Income Families (i.e., annual family income of less than $80,000) received grades that did not differ significantly from other students.

One issue of concern is that students could choose which version they took in Winter quarters before the Covid pandemic: that is, assignment of a student to a treatment was nonrandom. Disentangling the influence of format selection on student performance from the influence of course format itself proved challenging. We took several approaches to account for the influence of format selection, and some of them indicated that students’ choice of course format was a major factor in their grades (see Supplementary Materials: Format Selection).

When students could choose between course formats (Winter quarters before the pandemic), student demographics and average grades differed between formats. Students self-identifying as Women, seniors, and humanities majors disproportionately chose to enroll in the online version of the course (Fig. 1). Students during these quarters performed poorer in the online version, and notably humanities students and underrepresented minorities who enrolled in either the online or face-to-face formats received significantly lower grades than other students (Table S2).

One approach for disentangling the influence of student choice of format from those of course format was to conduct a well-controlled regression comparing the outcomes of students who chose the face-to-face version in the Winter quarters before the pandemic with those of students who took the course when only the online version was offered (i.e., Spring quarters before the pandemic). Total course grade (out of 100), when regressed on course format and on controls for student demographic and academic characteristics, indicated that course format had no significant effect on student performance (Table S3).

**Fig. 2:**
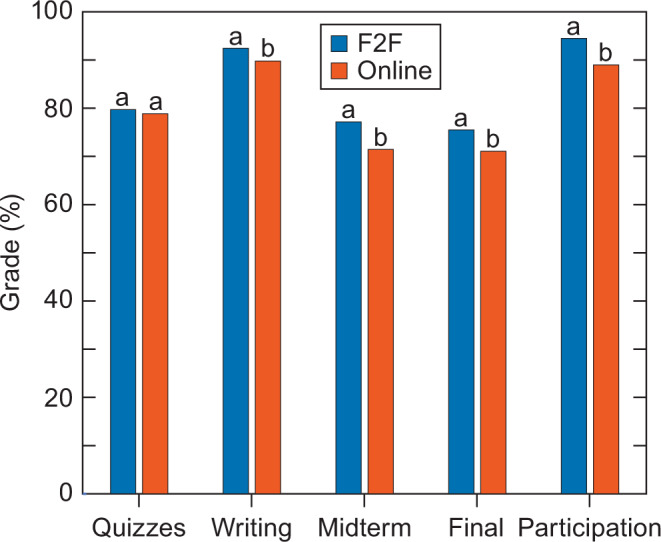
Grades (0 to 100) for various assessments during the Winter quarters before the pandemic when an introductory, undergraduate course on climate change was offered both face-to-face (F2F) and online. Different letters above the bars indicate that the grades on a type of assignment differed significantly (*P* < 0.01) between the students in the F2F and online versions.

Another approach for disentangling the influence of format selection from those of course format was to compare performance on different types of assessments. Weekly quizzes were administered online and based on the online textbook, and therefore depended entirely on online material, whereas the other assessments (weekly writing assignments, the midterm and final exams, and participation in weekly discussion sections) were probably enriched by face-to-face lectures and face-to-face discussion sections. During the Winter quarters before the pandemic, when students could choose between face-to-face and online versions, the scores on the quizzes did not differ significantly between the two formats, but scores on the other assessments were poorer for the student enrolled in the online version (Fig. 2). Moreover, students majoring in the humanities achieved lower scores on the quizzes, but course version influenced only the last quiz (one that focused on the sociology of climate change) (Table S4). These results indicate that the students who could choose the version of the course performed equally on material that was independent of course format but performed worse in the online version on material that depended on course format.

We based 10% of the overall course grade on participation in discussion sections, which we evaluated primarily on attendance. Discussion sections in the online version were conducted synchronously via video conferencing with up to 15 students per section, whereas discussion sections in the face-to-face version were conducted on campus with up to 25 students per section. Students enrolled in the online version of the course participated in 5.5% fewer discussion sections than those in the face-to-face version (Fig. 2). This was similar for a comparison of versions offered in the same quarter (Winter) and a comparison when the versions were offered in different quarters (Table S5).

## Discussion

This study offers both methodological and topical insights. We identified differences in outcomes between course formats using well-controlled regression analyses of various subsets of the data. The performance of a student in this course depended most strongly on the overall Grade Point Average of the student (Table 2, S1, S2, S3, and S5), indicating that this course required proficiency in the same skills as other college courses.

The outcomes of the students who only had an option of the online format (i.e., Spring quarters before the pandemic) did not differ significantly from those of the students who selected the face-to-face version in Winter quarters before the pandemic (Table S3). Perhaps our most illuminating findings were that differences in outcomes between formats were significant for the writing assignments and exams, but not the quizzes. We hypothesized that because the quizzes were based entirely on online material, online students would not be disadvantaged. This proved to be the case (Fig. 2). Poorer participation through lower attendance in the online discussion sections (Fig. 2) might be responsible for the poorer performance of online students on the writing assignments and exams (Fig. 2). Future research should seek approaches to address this deficiency in online courses.

This study’s findings offer insight into the effects of COVID-19 on higher education. Our results show that online coursework incurs only a small outcome penalty for students when a choice between formats is offered (Table 2). COVID-19 itself caused no significant difference in outcomes (Table S1). Given these findings, we are cautiously optimistic that an online format before or during COVID-19 may not be substantially detrimental to student learning in courses similar to the one studied here, if adequate care is given to aligning course content and instruction between formats.

One should consider the limitations of these findings as well. These data derived from an introductory, lower-division course that is usually taken as an elective, and these findings may not generalize to more technical or advanced courses, or courses that include lab work or group projects. Additionally, our use of well-controlled regression analysis to test the research questions limited our ability to account for selection. We discuss selection effects further in the supplemental materials.

Despite the hardship caused by the COVID-19 pandemic, it offers unique opportunities for further research on online course work. Many students are now enrolled in online courses that were previously face-to-face, providing much more data to compare online versus face-to-face outcomes, without the obfuscation of course format selection. We suggest that future research leverage these data for a better understanding of the efficacy of online coursework.

## Conclusions

Undergraduate students who chose the online version of an introductory course on climate change performed 2% worse (on a scale of 0–100) than those who chose the face-to-face version. The convenience of the online course—it required only one synchronous, online meeting per week versus three synchronous, on campus meetings per week—might be worth this small penalty. In particular, students who have to be away from campus classrooms for employment opportunities, family obligations, athletic events, year-abroad programs, or social distancing are well served by an online format.

## Materials and methods

Table 1 provides the syllabus for the course. During Winter quarters 2013 through 2015 and 2017 through 2020, the primary instructor (A. J. Bloom) taught both the online and face-to-face versions concurrently, whereas in Winter 2016, a second instructor (Dr. Margaret Swisher-Mantor) taught both versions. The primary instructor taught only the online version during Winter quarter 2021 and Spring quarters of 2013, 2014, 2015, 2017, 2018, and 2021.

All elements of the course are available for free at https://www.climatechangecourse.org/, including a free multi-media textbook at https://indd.adobe.com/view/7eafc24d-9151-4493-85d2-cb3f2e5a2a51 that is regularly updated. Before 2016, a printed version of the textbook was available for purchase (*23*). The course covered the (*a*) physical sciences (history of Earth’s climate, causes of change, and predictions), (*b*) biological consequences (direct effects of rising CO_2_, global warming, precipitation changes, and ocean acidification), (*c*) technological mitigation and adaptation, (transportation, electricity generation, buildings, and geoengineering), and (*d*) social sciences (economics, law, and social change). Lecture materials were available in 48 short (less than 15 min) videos or presented two times per week in live lectures of 50-minute duration that were streamed live over the internet or posted as videos on the course website on the same day. Students—be they enrolled in the online or face-to-face version of the course—had access to the lecture materials in all forms. Students had a weekly mandatory, 50-minute discussion section that met either synchronously online via video conferencing (Adobe Connect or Zoom) with up to 15 students per section with a choice of 8 different meeting times or face-to-face with up to 25 students per section with a choice of 4 different meeting times.

Assessments of the students included (*a*) weekly quizzes composed of 10 to 12 multiple choice questions drawn randomly from a pool of about 50 questions available as a practice quiz in the multi-media textbook, (*b*) participation in the weekly discussion sections based mostly on attendance, (*c*) weekly writing assignments that alternate between exercises and essays, (*d*) a proctored midterm exam with 25 multiple choice questions including those from the same question pools as the weekly quizzes and a few from the lectures and one essay question in which a student explained the greenhouse effect, and (*e*) a proctored final exam with 50 multiple choice questions including those from the same question pools as the weekly quizzes and a few from the lectures and one essay question in which a student explained what they would do, if anything, about climate change and why they would choose this course of action.

We predicted that performance on quizzes would not differ between students in the online and face-to-face versions of the course because the quizzes are based entirely on the textbook, whereas performance on the other assessments would be more dependent on course format because these rely more on information in lectures and discussion sections.

### Statistics

We fit the three models using an ordinary least squares linear regression (function *lm*) implemented in R version 4.0.3 (R Core Team, 2013):

1. *G*_*i*_ = *b*_0_ + *b*_1_(Format_*i*_ or Covid_*i*_) + *e*_*i*_,
2. *G*_*i*_ = *b*_0_ + *b*_1_(Format_*i*_ or Covid_*i*_) + *b*_2_MixedLang_*i*_ + *b*_3_NoneEngLang_*i*_ + *b*_4_Male_*i*_ + *b*_5_Senior_*i*_ + *b*_6_Humanities_*i*_ + *e*_*i*_,
3. *G*_*i*_ = *b*_0_ + *b*_1_(Format_*i*_ or Covid_*i*_) + *b*_2_MixedLang_*i*_ + *b*_3_NoneEngLang_*i*_ + *b*_4_Male_*i*_ + *b*_5_Senior_*i*_ + *b*_6_Humanities_*i*_ + *b*_7_GPA_*i*_ + *b*_8_URM_*i*_ + *b*_9_LowIncome_*i*_ + *b*_10_FirstGen_*i*_ + *e*_*i*_,

where *G*_*i*_ is the grade for student *i* on a 0 to 100 scale. In these models, the variable of interest is *Format* (a binary indicator coded as 1 for the online format and 0 for the face-to-face format) or *Covid* (a binary indicator coded as 1 for the course offerings in 2021 and 0 for the previous years). The coefficient *b*_1_ represents the marginal effect of the online course format or pandemic on grade. Model 1 yields a *b*_1_ value that represents the unconditional difference in mean student grade between the online and face-to-face versions of the course or the difference in mean student grade during the pandemic and before the pandemic. This value is an offset from *b*_0_, which represents the mean student grade in the face-to-face format or before the pandemic. Models 2 and 3 yield *b*_1_ values that represent the difference in mean student grade between the online and face-to-face versions of the course or the difference in mean student grade during and before the pandemic after accounting for demographic makeups and for previous academic achievement of the students in each format or each time period. The function *lm* calculated (*a*) ordinary least squares estimates of the coefficients (for COVID, Online, Mixed Lang. Home, Non-English Home, Male, Senior, Humanities, URM, Low Income, and First Generation designated to be 0 or 1 and for GPA which varied between 0 and 4) with standard errors, (*b*) *t* values for the Wald test of the hypothesis H_0_:*β*_i_ = 0, and (*c*) the associated *P* values. A *P* ≤ 0.05 was considered significant.

### Reporting summary

Further information on research design is available in the Nature Research Reporting Summary linked to this article.

## Supplementary information

Supplementary Information

Reporting Summary

## Data Availability

All materials for the course including the multi-media textbook are publicly available for free. Student grades and demographic information in the United States are confidential according to the FERPA (Federal Educational Rights and Privacy Act; https://www2.ed.gov/policy/gen/guid/fpco/ferpa/index.html). One can provide such data to the public only if aggregated for large groups (e.g., > 10 students). The authors judged that a dataset for large groups would duplicate the information already presented in Table 2, and S1–S7.
