# Supplementary material for: Remote learning slightly decreased student performance in an introductory undergraduate course on climate change
Source: Commun Earth Environ. 2022 Aug 6;3(1):177. doi: 10.1038/s43247-022-00506-6 (PMC9362672; doi:10.1038/s43247-022-00506-6)
Supplement: Supplementary file 1 — Supplementary Information [file 43247_2022_506_MOESM1_ESM.pdf]

Supplementary Materials for

Remote learning has a small but significant impact on student performance in an introductory undergraduate course on climate change

Sattik Ghosh, Stephanie Pulford, Arnold J. Bloom.

Correspondence to: ajbloom@ucdavis.edu

This PDF file includes:

Materials and Methods
Supplementary Text
Tables S1 to S7
Figure S1
Supplementary References

Supplementary Materials and Methods

Influence of Student Format Selection

We took several approaches to account for the influence of student choice of format. One was propensity-weighted regressions that aim to equalize characteristic differences in the treatment group (online version) and control group (face-to-face version). A full description of this analysis is provided in a section "Accounting for Selection Bias" below. The propensity weights failed to reduce the difference in survey responses (Table S6), indicating that propensity-weighted regressions were not appropriate for this study.

A second survey-based approach was to compare student attitudes toward and experience with online courses at the start of several course offerings. Students were surveyed for two concurrent course offerings in Winter 2019 (i.e., one face-to-face and one online) and online course offerings in Winter 2021 and Spring 2021 when because of the pandemic, there were no comparable face-to-face offerings. Only complete survey responses were included for these analyses. We conducted a set of three Mann-Whitney-Wilcoxon comparison tests [1] to identify differences in summed survey scores between:

1. Students who selected into the online format in Winter 2019 (n = 49) versus students who selected into the face-to-face format in Winter 2019 (n = 42),
2. Students who took the pandemic-induced online formats of the course in Winter and Spring 2021 (n = 197) versus students who selected into the online format in Winter 2019, and
3. Students who took the pandemic-induced online formats of the course in Winter and Spring 2021 versus students who selected into the face-to-face format in Winter 2019.

Results of these comparison tests are presented in Table S7. Because survey scores between the pandemic-induced online students in 2021 and the Winter 2019 face-to-face students were similar, we infer that the two sets of students had similar opinions about online coursework. Differences in grades between these students, therefore, derived from course format rather than student selection. An analysis for Winters 2013-2020 F2F versus Spring 2013-2019 online showed no difference in grades (Table S3). Moreover, an analysis of 2013-2020 online versus Winter & Spring 2021 (pandemic online) also showed no difference in grades (Table S1).

Another approach for disentangling the effects of student choice of format from those of course format was to conduct a well-controlled regression comparing the outcomes of students who chose the face-to-face version in the Winter quarter with those of students who took the course in when only the online version was offered (i.e., Spring quarters before the pandemic or Winter and Spring quarter during the pandemic). Total course grade (out of 100), when regressed on course format and controls for student demographic and academic characteristics, indicated that course format had no significant effect on student performance (Table S3). Of course, other factors related to time of year (e.g., thoughts of love or thoughts of graduation during the spring or the amount of sunshine) or the conditions of the pandemic (e.g., more experience with online courses) could be responsible.

A fourth approach for disentangling the effects of format selection from those of course format was to compare performance on different types of assessments. Weekly quizzes were administered online and based on the online textbook, and therefore depended entirely on online material, whereas the other assessments (weekly writing assignments, the midterm and final

exams, and participation in weekly discussion sections) were likely enriched by face-to-face lectures and face-to-face discussion sections. During the Winter quarters before the pandemic when students could choose between online and face-to-face versions, the scores on the quizzes did not differ significantly between the two formats, but scores on the other assessments were poorer for the student enrolled in the online version (Fig. 2). Moreover, students majoring in the humanities achieved lower scores on the quizzes, but for all students the course version had a significant effect only in the last quiz that covered the sociology of climate change (Table S4). These results indicate that the students who could choose the version of the course performed equally on material that was independent of course format but performed worse in the online version on material that depended on course format.

Accounting for Selection Bias

While controlling for previous achievement and for demographic differences between the formats provides useful context to identify the effect of the online format on grades, it is possible that unobservable traits related to success in the course are driving students' decisions to enroll in one format over the other. A common approach to address this selection issue is to apply propensity-weighted or propensity-matched regressions. These approaches equalize characteristic differences among the students in the control (face-to-face version) and treatment (online version) groups. The underlying assumption here is that these differences are related to selection; that is, some types of students are more prone to taking the online format than others.

Propensity weighted regressions include an estimation step and an application step [2]: The estimation step regresses the treatment group indicator on demographic covariates related to selection. This regression estimates *propensity scores* (PS)—values that correspond to the subject's probability of being in the treatment group, conditional on the selection covariates—for each subject. These scores are converted into weights according to Equations 1 and 2 [3].

(1) $w = \frac{1}{PS}$ for students in the treatment group

(2) $w = \frac{1}{1-PS}$ for students in the control group

Higher weights correspond to smaller probabilities that a subject is in their observed group. Here, we assigned a large weight to a face-to-face format student who exhibits demographic characteristics that more resemble those of a typical online student. The goal of applying these weights to all observations is to create treatment and control groups that are balanced on these demographic characteristics. To ensure that balance was achieved, we regressed each covariate on course format with propensity weights applied. In these regressions, non-significant relationships between the covariates and course format indicate balance between the groups.

In the application step, we applied the weights generated in the estimation step to regressions of the outcome on the treatment. Because the weighting process balances selection factors between the treatment and control groups, coefficients on the treatment variable may at this stage be interpreted as the causal effect of the treatment variable on the outcome. Controls for other variables that possibly may be related to the outcome via mechanisms separate from student selection may also be included here.

We regressed course format on English Language Learner (ELL) status, self-identified gender, senior class standing, and discipline of major (Table 2). ELL status, gender, and senior class standing were significant factors in predicting whether a student might choose the online format, whereas discipline was not. This result was used to estimate a propensity score for each student—that is, each student was assigned a value corresponding to their probability of being in

the online course format conditional on their ELL status, gender, senior class standing, and discipline. These values were converted into weights according to Equation 1.

We then leveraged survey data to validate the propensity weights produced in the first step of the regression. The survey posed students with questions related to their attitude toward online courses. The expectation was that students who responded positively to online course in the survey were more likely to have selected into the online course. Because survey responses more directly measure selection factors, they offer an opportunity to validate the generated propensity weights. We applied the propensity weights to a regression of course format based on survey response. Substantial reduction in the association between course format and survey response after applying propensity weights would provide evidence that the propensity weights truly reflect selection factors.

Next, we aimed to verify that these propensity weights corresponded with actual attitudes toward online courses in a survey where students rated their agreement with statements about online courses on a scale from 1 (strongly disagree) to 7 (strongly agree). We posed all questions so that agreement would indicate favor toward online courses.

Summed survey scores were regressed on the indicator for online enrollment. We then performed the same analysis with propensity weights applied. The average score on the survey was 77.75 (out of 182) in the face-to-face version of the course and was 14.93 points higher in the online format (Table S6). These scores did not appreciably change when propensity weights were applied: after adjusting for differences in distributions of ELL status, gender, senior class standing, and discipline between the two formats, students in the online format still showed more positive responses toward online courses than students in the face-to-face format. Because this propensity weighting approach does not seem to account for attitudinal differences (i.e., selection factors) toward online courses between students in each course format, we proceeded to employ a well-controlled regression analysis without propensity weighting.

Table S1. Regressions of course grade for the online version of an introductory, undergraduate course on climate change before and during the Covid pandemic.

Variable	Model 1	Model 2	Model 3
Intercept	83.70***	86.06***	48.48***
Covid	0.88	0.86	–0.59
Mixed Lang. Home		–0.29	1.93*
Non-English Home		–0.45	0.22
Male		–1.39	–0.50
Senior		–1.73	–2.54**
Humanities		–4.80***	–6.84***
GPA			12.76***
URM			–5.84***
Low Income			–1.64
First Gen			1.27

"Intercept" predicts the average grade of students on a 0 to 100 scale. The three models include Model 1 where "Covid" (0 or 1) is the influence of Covid on a student's grade. Model 2 adds the influence of other Languages being spoken at home, self-identifying as Male, being a Senior, and majoring in Humanities (all 0 or 1). Model 3 adds the influence of the student's GPA (grade point average between 0 and 4), being an Underrepresented Minority (0 or 1) (African Americans, American Indian/Alaska Native, Chicanx/Latinx including Puerto Rican, and Pacific Islander including Native Hawaiian), being from a Low-Income family (0 or 1) (annual family income of less than $80,000), and being the First Generation to attend college (0 or 1). Asterisks following a number indicates *P*-values associated with *t* values for the Wald test of the hypothesis H_0:β_i=0: "*" indicates $P < 0.05$, "**" $P < 0.01$, and "***" $P < 0.001$

Table S1. Regressions of course grade for the online version of an introductory, undergraduate course on climate change before and during the Covid pandemic.

Table S2. Regressions of course grade in an introductory, undergraduate course on climate change for the Winter Quarters before the Covid pandemic in which both face-to-face and online versions were offered.

Variable	Model 1	Model 2	Model 3
Intercept	85.93***	88.22***	51.93***
Online	–3.04***	–3.23***	–3.02***
Mixed Lang. Home		–0.21	1.73*
Non-English Home		–0.82	0.01
Male		–1.83*	–0.95
Senior		–0.35	–1.08
Humanities		–4.85***	–6.71***
GPA			11.80***
URM			–2.90***
Low Income			–0.11
First Gen			1.26

"Intercept" predicts the expected grade on a 0 to 100 scale of students in the reference group in each model (i.e., a face-to-face student with values of 0 on all included control variables). The three models include Model 1 where "Online" (0 or 1) is the influence of the online version on a student's grade. Model 2 adds the influence of other Languages being spoken at home, self-identifying as Male, being a Senior, and majoring in Humanities (all 0 or 1). Model 3 adds the influence of the student's GPA (grade point average between 0 and 4), being an Underrepresented Minority (0 or 1) (African Americans, American Indian/Alaska Native, Chicanx/Latinx including Puerto Rican, and Pacific Islander including Native Hawaiian), being from a Low-Income family (0 or 1) (annual family income of less than $80,000), and being the First Generation to attend college (0 or 1). Asterisks following a number indicates *P*-values associated with *t* values for the Wald test of the hypothesis H_0:β_i=0: "*" indicates $P < 0.05$, "**" $P < 0.01$, and "***" $P < 0.001$

Table S2. Regressions of course grade in an introductory, undergraduate course on climate change for the Winter Quarters before the Covid pandemic in which both face-to-face and online versions were offered.

Table S3. Regressions of overall course grade in an introductory, undergraduate course on climate change for face-to-face students in Winter quarters before the pandemic versus those for online students in Spring quarters before the pandemic.

Variable	Model 1	Model 2	Model 3
Intercept	86.78***	87.58***	68.27***
Online	–0.70	0.14	–0.47
Mixed Lang Home		–0.67	0.93
Non-English Home		–2.32**	–0.88
Male		0.50	0.83
Senior		–2.55**	–2.77**
Humanities		–0.06	–0.99
GPA			6.40***
URM			–3.68***
Low Income			–1.06
First Gen			0.74

"Intercept" predicts the expected grade on a 0 to 100 scale of students in the reference group in each model (i.e., a face-to-face student with values of 0 on all included control variables). The three models include Model 1 where "Online" (0 or 1) is the influence of the online version on a student's grade. Model 2 adds the influence of other Languages being spoken at home, self-identifying as Male, being a Senior, and majoring in Humanities (all 0 or 1). Model 3 adds the influence of the student's GPA (grade point average between 0 and 4), being an Underrepresented Minority (0 or 1), being from a Low-Income family (0 or 1) (annual family income of less than $80,000), and being the First Generation to attend college (0 or 1). Asterisks following a number indicates P-values associated with t values for the Wald test of the hypothesis $H_0{:}\beta_i=0$: "*" indicates $P < 0.05$, "**" $P < 0.01$, and "***" $P < 0.001$

Table S3. Regressions of overall course grade in an introductory, undergraduate course on climate change for face-to-face students in Winter quarters before the pandemic versus those for online students in Spring quarters before the pandemic.

Table S4. Regression of grades on various quizzes in the Winter quarters of an introductory, undergraduate course on climate change offered both online and face-to-face.

Variable	Quiz 1	Quiz 2	Quiz 3	Quiz 4	Quiz 5
Intercept	77.99***	76.80***	76.73***	80.86***	88.98***
Humanities	–2.74	–4.18	–0.52	–4.52	–7.50**
Online	0.29	0.37	1.01	–1.08	–1.03
Humanities × Online	0.71	2.24	1.19	–2.42	2.77
**Variable**	**Quiz 6**	**Quiz 7**	**Quiz 8**	**Quiz 9**	**Quiz 10**
Intercept	76.15***	90.73***	88.76***	75.71***	67.09***
Humanities	–5.67*	–2.68	–4.04	–2.72	–2.20
Online	0.18	-2.48	–4.36*	–0.73	3.84*
Humanities × Online	4.82	–0.75	–1.81	–1.91	1.38

Quizzes 1 through 4 focused on the physical science bases of climate change, Quiz 5 focused on the consequences of climate change for the biosphere, Quiz 6 and 7 focused on mitigation strategies for climate change, Quiz 8 focused on the economics of climate change, Quiz 9 focused on international regulation of climate change, and Quiz 10 focused on the sociology of climate change. Asterisks following a number indicates *P*-values associated with *t* values for the Wald test of the hypothesis H_0:β_i=0: "*" indicates $P < 0.05$, "**" $P < 0.01$, and "***" $P < 0.001$

Table S4. Regression of grades on various quizzes in the Winter quarters of an introductory, undergraduate course on climate change offered both online and face-to-face.

Table S5. Regressions of participation in discussion sections (on a scale of 0 to 100) based primarily on attendance. Comparisons of face-to-face students in Winter quarters versus those for online students in Spring quarters (F2F W vs. Online S) or in the face-to-face version versus the online version in Winter Quarters (F2F W vs. Online W).

Variable	F2F W vs. Online S	F2F W vs. Online W
Intercept	80.76***	78.52***
Online	–3.03*	–3.54**
Mixed Lang. Home	1.22	0.31
Non-English Home	0.16	–2.17
Male	0.13	–1.05
Senior	–5.65***	–3.43**
Humanities	–1.20	–4.75***
GPA	4.96***	5.71***
URM	–1.80	–1.04
Low Income	–1.24	–0.73
First Gen	–0.27	2.79*

"Intercept" predicts the expected grade of students in the face-to-face version. "Online" was the influence of the online version on a student's grade. We included only students who continued to submit work after the fourth week. "Mixed Lang. Home" was the influence of other Languages spoken at home, "Non-English Home" was the influence of Non-English being the principal language at home, "Male" being self-identified as male, "Senior" being in the last year of college, and "Humanities" majoring in a Humanities discipline, "GPA" was grade point average, "URM" being an Underrepresented Minority, "Low Income" being from a Low Income family (annual family income of less than $80,000), and "First Gen" being the First Generation to attend college. Asterisks following a number indicates *P*-values associated with *t* values for the Wald test of the hypothesis $H_0:\beta_i=0$: "*" indicates $P < 0.05$, "**" $P < 0.01$, and "***" $P < 0.001$

Table S5. Regressions of participation in discussion sections (on a scale of 0 to 100) based primarily on attendance. Comparisons of face-to-face students in Winter quarters versus those for online students in Spring quarters (F2F W vs. Online S) or in the face-to-face version versus the online version in Winter Quarters (F2F W vs. Online W).

Table S6. Differences in unweighted and weighted regressions of scores in a survey about student attitudes about course formats. "Intercept" predicts the average survey rating of students in the face-to-face version. "Online" predicts the influence of the online version on a student's rating. Asterisks following a number indicates significance with "***" = $P < 0.001$.

	Model	
Variable	Unweighted	Weighted
Intercept	77.75***	77.94***
Online	14.93***	14.80***

Table S6. Differences in unweighted and weighted regressions of scores in a survey about student attitudes about course formats. "Intercept" predicts the average survey rating of students in the face-to-face version. "Online" predicts the influence of the online version on a student's rating. Asterisks following a number indicates significance with "***" = $P < 0.001$.

Table S7. Mann-Whitney-Wilcoxon comparison tests of online favorability scores. Asterisks following a number indicates significance with "***" = *P* < 0.001.

Comparison (Group 1 vs. Group 2)	Mean Group 1	Mean Group 2	*W*-statistic	*P*-value
W19 Online vs. W19 F2F	92.90	78.61	363	0.00***
W19 Online vs. W21/S21 COVID-19 Online	92.90	78.03	7493.5	0.00***
W19 F2F vs. W21/S21 COVID-19 Online	78.61	78.03	4170	0.94

Table S7. Mann-Whitney-Wilcoxon comparison tests of online favorability scores. Asterisks following a number indicates significance with "***" = *P* < 0.001.

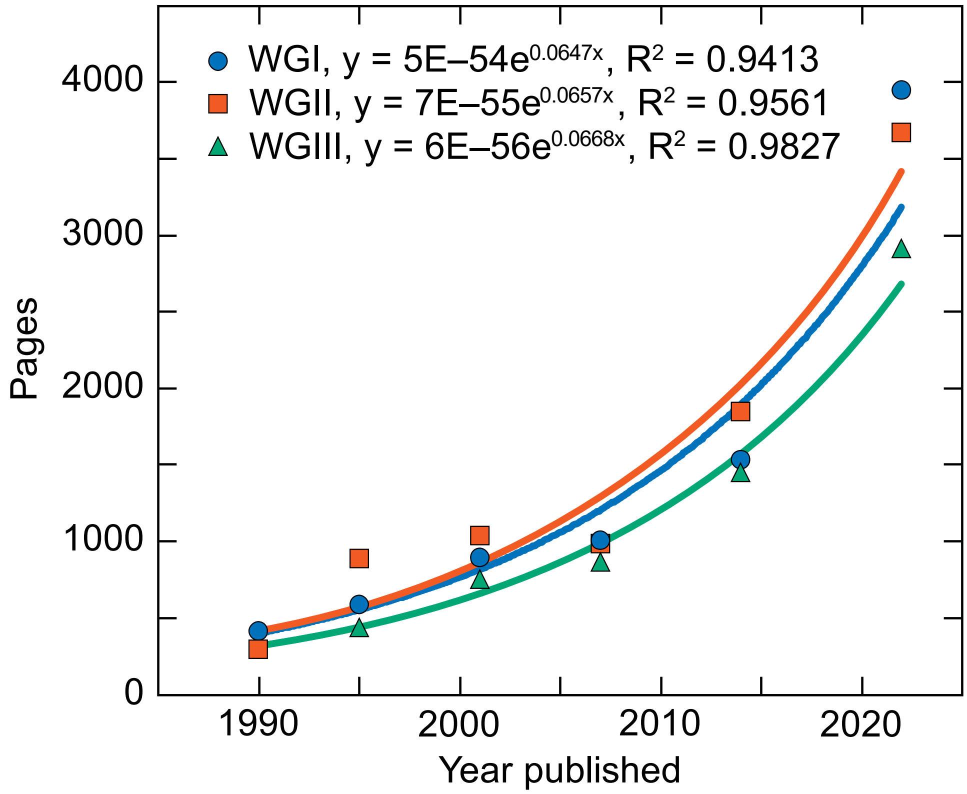

Fig. S1. Number of pages versus year published for the assessment reports of the three Working Groups (WGI emphasizes physical science underpinnings, WGII emphasizes impact and adaptation, and WGIII emphasizes mitigation) of the Intergovernmental Panel on Climate Change. Values are for an exponential trend in the data.

Supplementary References

1 de Winter, J. F. & Dodou, D. Five-point likert items: t test versus Mann-Whitney-Wilcoxon (Addendum added October 2012). *Practical Assessment, Research, and Evaluation* **15**, 11, doi: https://doi.org/10.7275/bj1p-ts64 (2010).

2 Harder, V. S., Stuart, E. A. & Anthony, J. C. Propensity score techniques and the assessment of measured covariate balance to test causal associations in psychological research. *Psychological Methods* **15**, 234 (2010).

3 Olmos, A. & Govindasamy, P. Propensity scores: a practical introduction using R. *Journal of MultiDisciplinary Evaluation* **11**, 68-88 (2015).
